# Chevalier Jackson: father of endoscopic surgery, and champion of women in medicine, social justice, and public health

**DOI:** 10.1007/s00464-023-10256-x

**Published:** 2023-07-13

**Authors:** Sven E. Eriksson, Blair A. Jobe, Shahin Ayazi

**Affiliations:** 1 Foregut Division, Surgical Institute, Allegheny Health Network, Pittsburgh, PA USA; 2 Chevalier Jackson Research Fellowship, Esophageal Institute, Western Pennsylvania Hospital, Allegheny Health Network, 4815 Liberty Avenue, Suite 439, Pittsburgh, PA 15224 USA; 3 Department of Surgery, Drexel University, Philadelphia, PA USA

**Keywords:** Chevalier Jackson, Endoscopy, Bronchoscopy, History of medicine, Foreign body, Women in medicine

## Abstract

**Backgrounds:**

Chevalier Jackson (1865–1958) was a pioneering force in the medical world, whose extraordinary contributions to surgery and public health have left an indelible impact. He developed the endoscope and perfected the bronchoscope, and his mastery of these tools enabled him to transform the prognosis of foreign body aspiration from 98% mortality to 98% survival. He was also a passionate advocate of public health chairing the national committee on lye legislation, which culminated in the Caustic Poison Act, responsible for poison and antidote labels. Yet Jackson’s accomplishments were not limited to these. The aim of this manuscript was to shed light on Chevalier Jackson’s lesser-known contributions to surgical science and culture, and to celebrate and honor the life of this remarkable surgeon.

**Methods:**

Digital and physical historical records from the National Library of Medicine, Smithsonian Institution, Heinz History Center in Pittsburgh PA, and Sunrise Mill Museum, Montgomery County PA were reviewed for Chevalier Jackson’s scientific, cultural, and social contributions to the field of surgery.

**Results:**

Among his lesser-known contributions, Chevalier Jackson was the first to describe erosive esophagitis. He developed the first standardized tracheotomy procedure, still in use today. He was ahead of his time in many ways, pioneering a multidisciplinary approach to medicine, advocating for patient-centered care, and advancing the inclusion of women in the medical profession.

**Conclusion:**

Chevalier Jackson’s legacy extends far beyond the tools and techniques he invented. He was a champion of social justice, a protector of patients, and an inspiration to medical professionals across the globe.

For more than a century Fellows of the American College of Surgeons have pledged themselves to the art and science of surgery, making a commitment to uphold certain ideals of professionalism, including integrity, inclusivity, compassion, service to the patient, advocacy for public health and advancement of the profession. All surgeons strive to uphold these high traditions; however, in the last 150 years few have exemplified this ethos quite as well as Chevalier Jackson (1865–1958). Born in Pittsburgh, Pennsylvania in 1865, the year the American Civil War ended, Chevalier Jackson would go on to become one of the most renowned surgeons in the world. He became known as a pioneer in laryngology, the developer of the esophagoscope, the perfector of the bronchoscope, the savior of more than 2000 patients who inhaled or swallowed foreign bodies, a prolific author with over 700 articles and 12 textbooks to his name, a globally recognized surgical educator, a founding member and inaugural governor of the American College of Surgeons and the father of endoscopic surgery [1–3]. In this manuscript we pay tribute to his exemplary scientific contributions and shed light on his character and his lesser known contributions to public health, medical education, social justice and service to the profession of medicine, most notably in how he championed the inclusion of women and people of color as physicians.

## Pioneer, innovator, surgeon

**Fig. 1:**
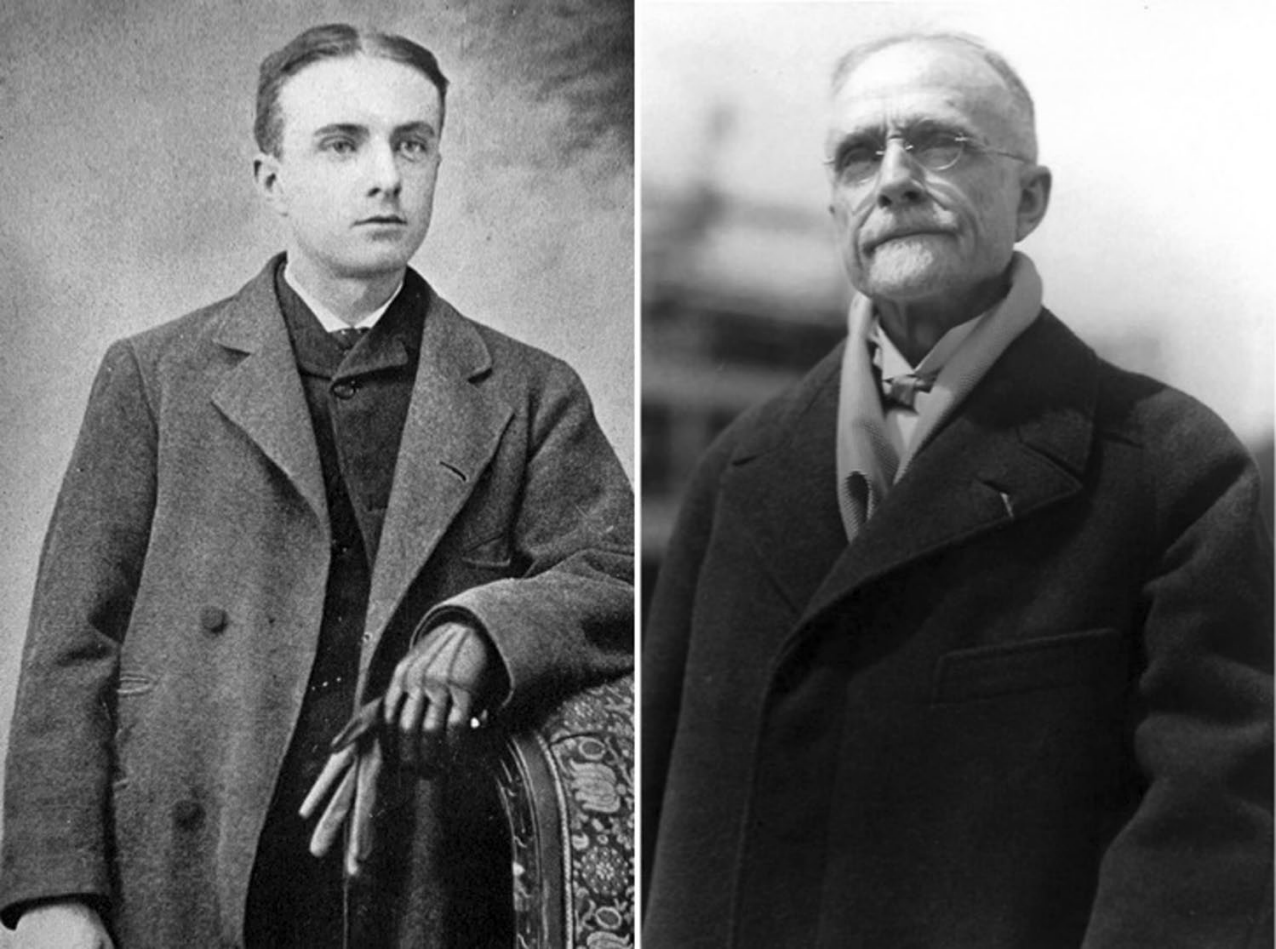
Chevalier Quixote Jackson MD FACS, at age 21, one-year after starting his practice in Pittsburgh at Western Pennsylvania Hospital (Left), and during his tenure as department chair at all 5 medical colleges in Philadelphia (Right). *Photos obtained from The Life of Chevalier Jackson: An Autobiography. New York: The MacMillan Company; 1939. (Left) and Chevalier Jackson papers. 1890–1964. Located in: Modern Manuscripts Collection, History of Medicine Division, National Library of Medicine, Bethesda, MD; MS C 292. (Right)*

Chevalier Jackson showed evidence of original thinking and problem solving from the beginning of his career. After graduating from Western Pennsylvania University (now University of Pittsburgh) at just 17 years old, Jackson attended Jefferson Medical College in Philadelphia (Fig. 1). During medical school he had the opportunity to read the complete works of Sir Morell Mackenzie (1837–1892) and, ever driven by the “intrigue of the impossible,” was inspired to specialize in laryngology at the phrase “it was impossible to see the larynx of some patients” [2]. At the time, nearly all physicians were in general practice, with Ophthalmology being one of the only recognized specialties. Chevalier Jackson received strong warnings against specialization from his friends and colleagues, concerned he would never be able to attract enough patients with a narrow focus on laryngology. Nevertheless, he established himself as a successful specialist in Pittsburgh by staunchly referring all general medicine cases to other local physicians, thus earning their respect, appreciation, and reciprocation. In doing so he engendered a culture of interdisciplinary cooperation, a given in routine modern practice, but a novel concept at the time. Furthermore, Jackson was an early proponent of multidisciplinary collaboration in patient care. At the 1930 Bronchoscopic Society meeting he delivered a speech on his novel use of bronchoscopically planted radium needles for the radiotherapeutic treatment of lung cancer, during which he submitted that treatment required a multidisciplinary approach, a concept decades ahead of its time. It “is not a one-man job” he said, suggesting practice be shared among the general practitioner, bronchoscopist and surgeon, foreshadowing the modern cooperation between oncology, radiation oncology and surgical oncology today [4].

**Fig. 2:**
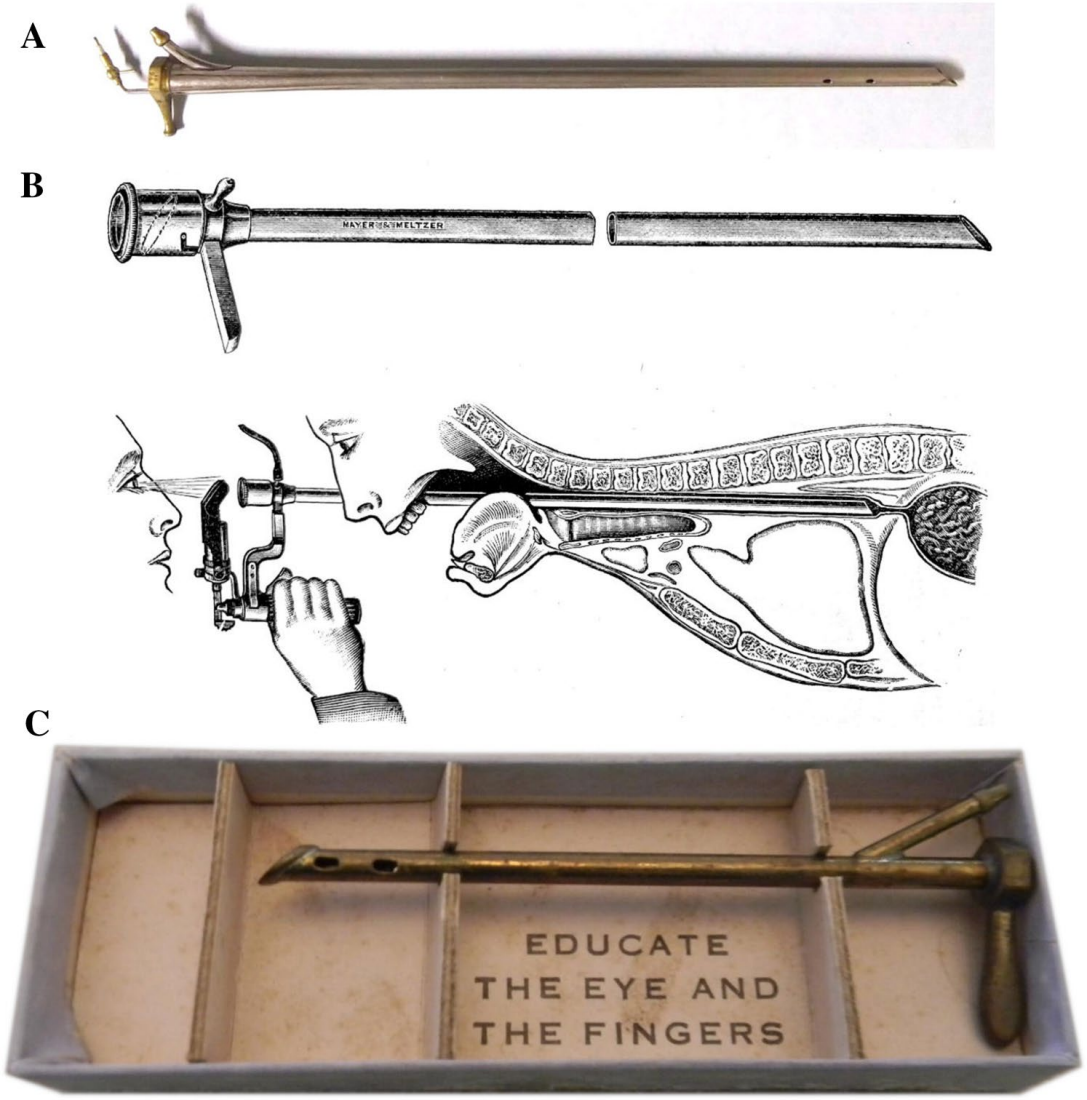
Chevalier Jackson’s endoscope (**A**). Illustrations of the essential parts and direct vision inflating with a Brunnings handle lamp (**B**). Each instrument was shipped in a box with the mantra Chevalier learned from his father and shared with his trainees: “Educate the Eye and the Fingers” (**C**). *Photos obtained from Chevalier Jackson papers. 1890–1964. Located in: Modern Manuscripts Collection, History of Medicine Division, National Library of Medicine, Bethesda, MD; MS C 292*

**Fig. 3:**
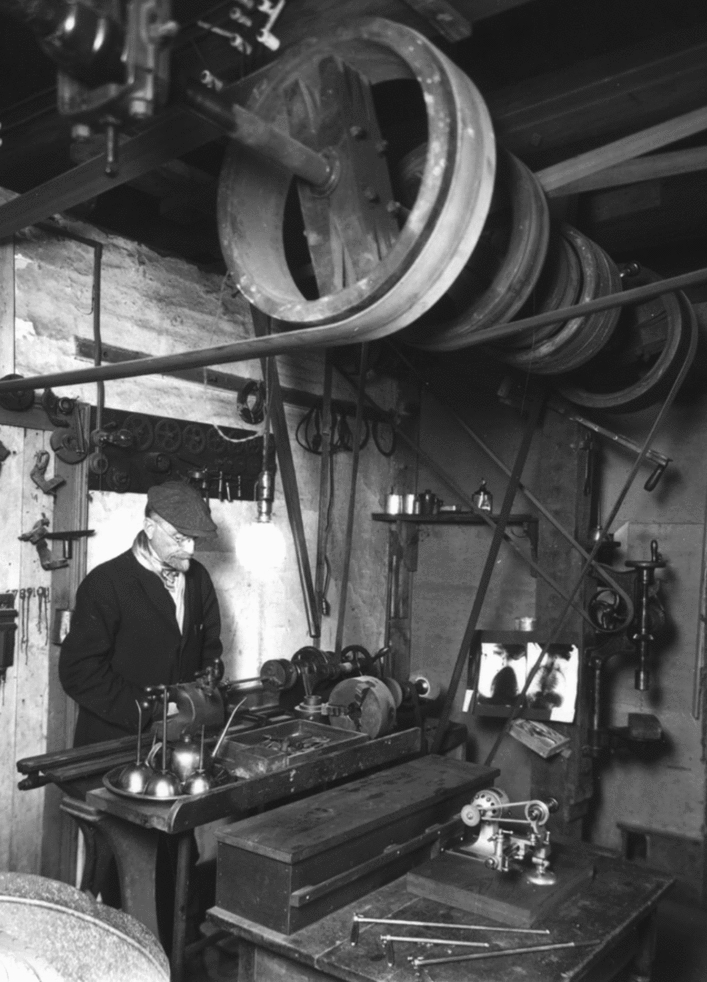
Although, the initial prototypes for his endoscopes were built in a Pittsburgh workshop owned by one of his friends and a machinist, Andrew Lascher, once he moved to Sunrise Mill outside Philadelphia, Jackson used the gristmill as a workshop and art studio, as seen here building several different sized bronchoscopes, which are sitting on the table in the foreground. *Photo obtained from The Life of Chevalier Jackson: An Autobiography. New York: The MacMillan Company; 1939*

In 1886, during a trip to the United Kingdom to learn directly from Sir Morell Mackenzie, Chevalier Jackson observed a crude and impractical esophagoscope of Mackenzie’s design. Mackenzie’s esophagoscope consisted of a thin long speculum separated and attached by rings, forming a skeleton tube. Illumination was achieved through reflection off an angulated mirror at the proximal end of the instrument. However, this “esophagoscope” could not be inserted past the distal pharynx [5]. Upon his return to Pittsburgh, Chevalier Jackson began to work on his own design. He had the benefit of 80 years of efforts to visually inspect the esophagus. In 1806, Philipp Bozzini (1773–1809) introduced the “Lichtleiter” (light conductor), the first instrument designed to illuminate the cavities of the body, capable of illuminating the proximal pharynx. The urologist Antoin Jean Desormeaux (1815–1894) developed the first urethroscope based on Bozzini’s design in 1853 and coined the term “endoscopy.” After studying the technique of a sword swallower, Adolf Kussmaul (1822–1902) passed a modified Desormeaux endoscope into the thoracic esophagus and diagnosed an esophageal carcinoma [5]. Finally, in 1890 Chevalier Jackson was able to craft an endoscope “worthy of its name” (Fig. 2). Illumination was initially provided by a headlight. Edison had invented the light bulb 11 years prior, but initial designs were too bulky and generated too much heat for use inside the esophagus. Henry Koch (1851–1915) an urologist, and electrician Charles Preston, both from Rochester, New York solved this problem by inventing the Mignon bulb, a replaceable miniaturized low-amperage bulb for use in a urethroscope. In 1902 Max Einhorn (1862–1953) suggested Jackson to utilize a distal light source, thus, “putting the light where the problem is.” The Chevalier Jackson endoscope became the first to incorporate a channel for a distal light source, with a separate channel for suction, and a groove for instrumentation in addition to visualization (Fig. 3**).** Armed with this tool, Jackson introduced a paradigm shift from mere inference to direct examination of the esophagus and stomach and was able to expand his practice into foreign body removal. However, endoscopy was not an immediate success. In his haste to share his development with the wider medical community, Chevalier Jackson neglected to warn would-be endoscopists of the procedure’s technical demands. Several physicians attempted endoscopy with disastrous outcomes, and endoscopy was condemned as the “hobby of an enthusiast.” Dismayed, Jackson informed his instrument maker that the endoscope was not ready for distribution and abstained from publishing any further findings for 15 years [2].

The problem of premature promotion of the endoscope was a learning experience, which prompted Chevalier Jackson to develop rigorous safety and educational protocols for endoscopic foreign body retrieval. He would first determine whether an instrument was capable of retrieving a given foreign body by placing and retrieving a replica of the object in a rubber tube. Then he would determine the safety of the instruments and removal techniques in dynamic tissues by placing and removing the object in an anesthetized dog. Only with success at this stage would there be confidence that the foreign body could be safely and effectively removed from the patient [6]. In his later years he employed this same safety protocol to educate endoscopic surgeons across the world. He held several educational courses in France, where experimentation on healthy human subjects was permitted, yet teaching using an animal model was always mandatory before trainees were permitted to operate on human patients [7].

**Fig. 4:**
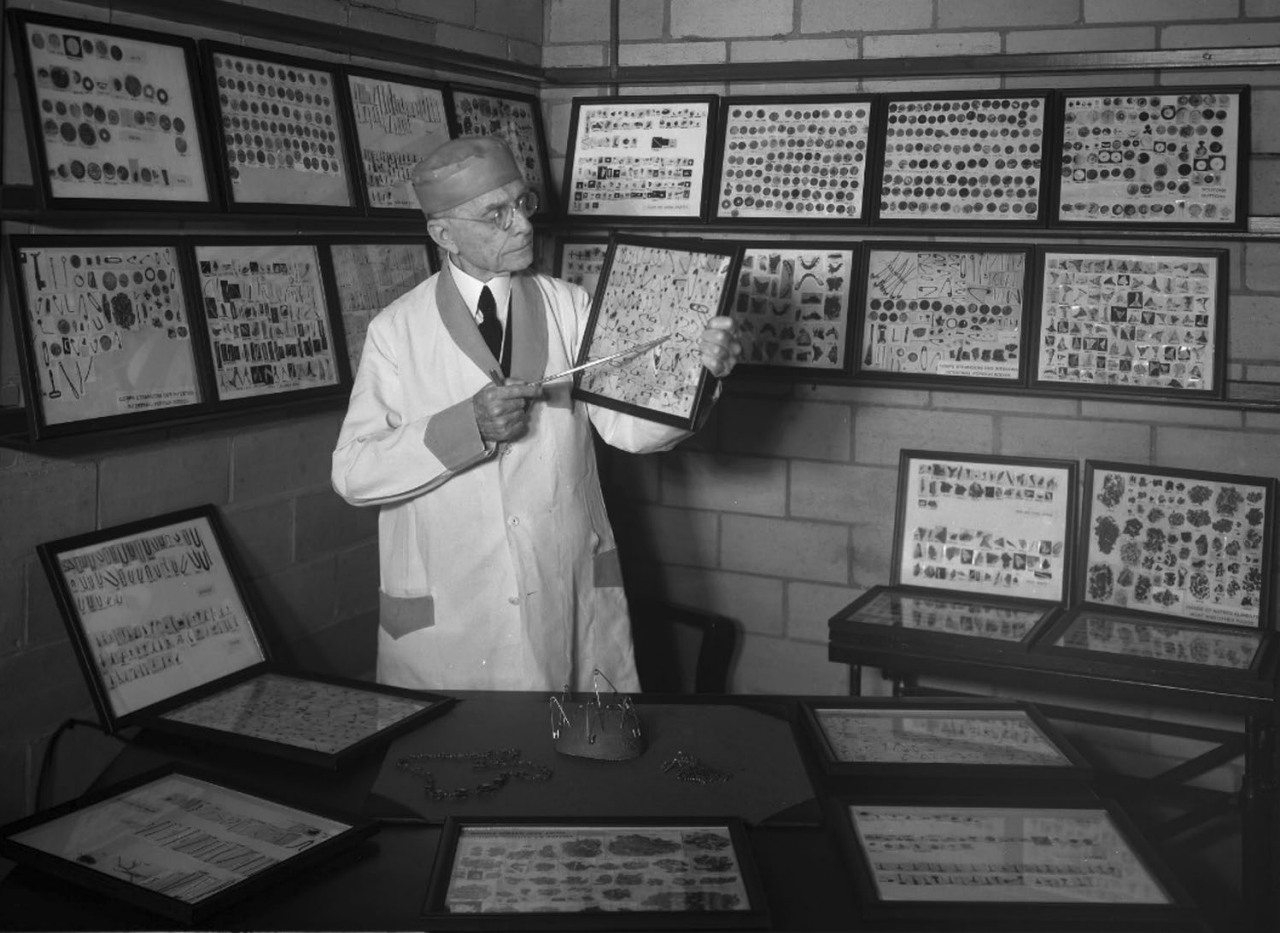
Chevalier Jackson and the collection of 2,374 foreign bodies he personally removed from the airway and esophagus of patients.* Photos obtained from Chevalier Jackson papers. 1890–1964. Located in: Modern Manuscripts Collection, History of Medicine Division, National Library of Medicine, Bethesda, MD; MS C 292*

Every foreign body removal was carefully cataloged, documenting the nature of the object, its location, and the method of its retrieval. He began proactively studying, placing, and removing various objects to master the technique for future patients. Preparation was the key to his success, often expressing the aphorism “2 min preparation, 2 h surgery; 2 h preparation, 2 min surgery” [8]. Chevalier Jackson became so proficient that his assistants often remarked that he could remove foreign bodies faster than they could place them. The only time his assistant, Dr. Isaac Hope Alexander (1879–1958), stumped the master was when he placed an open safety pin with the needle point up [8]. That day Chevalier Jackson designed and manufactured the solution, an endoscopic instrument with a dynamically angled distal ring, the diameter of which was equal to the width of the closed safety pin. The endoscope was used to protect the sharp end of the needle while the ring was inserted distal to the safety pin. A separate endoscopic grasper was used to push the safety pin into the ring, closing the pin so that it could be removed without injury. A wide array of specialized graspers were designed for endoscopic surgery, including the alligator forceps, which was the most frequently used instrument in foreign body removal [9]. Throughout his career Chevalier Jackson developed a collection of 2374 foreign bodies he had removed from the airway or esophagus of his patients, which is currently housed in the Mutter Museum in Philadelphia (Fig. 4).

Foreign body removal may have been the procedure that Jackson initially became well known for, but he was responsible for a number of breakthroughs with the endoscope. He was the first to identify erosive esophagitis as a pathological condition. Until his landmark paper in 1929, esophagitis and ulceration had only been reported on autopsy and was thought to be a postmortem condition. He also correctly linked the symptom of heartburn and the development of esophagitis with the then purely theoretical retrograde flow of acidic gastric contents into the distal esophagus [10]. In addition to being the first to meticulously catalog, describe, illustrate, and publish the endoscopic appearance of a wide array of esophageal pathology, he developed the first endoscopically-assisted surgeries. One such operation was the endoscopically-assisted, one-stage transcervical diverticulectomy for Zenker’s diverticulum. In early practice, aspiration pneumonia and mediastinal sepsis were common after diverticulectomy due to the stagnant material inside the diverticulum. To mitigate these complications a two-stage surgery was popularized: the diverticulum was first elevated and allowed to empty by gravity over a few weeks before resection. Jackson’s one-stage procedure improved the morbidity and mortality of this procedure in a number of ways: (1) direct visualization of the diverticular sack enabled emptying and washout of stagnant contents immediately before resection, reducing risk of infection, aspiration pneumonia and a second operation; (2) transillumination from the endoscope helped identify the diverticulum without extensive cervical exploration and reduced operative times; and (3) the endoscope could act as a bougie in the true lumen, reducing the likelihood of iatrogenic stricture [11]. This innovative operation was an essential step in the evolution of the modern management of Zenker’s diverticulum, which is now frequently performed entirely endoscopically.

Chevalier Jackson had been practicing direct laryngoscopy and endoscopy for several years by the time Dr. Gustav Killian successfully removed a bone from the bronchus of a farmer, earning the title ‘father of bronchoscopy’ in 1897 [5]. Inspired by Killian’s reports, Chevalier Jackson began developing his own bronchoscopes and bronchoscopic techniques. He adapted the safety and efficacy protocols he developed with endoscopy and began to successfully remove aspirated foreign bodies from the bronchial tree. At the time, if a foreign body could not be coughed up, it was inevitably fatal due to asphyxiation or indolent sequelae of a chronically obstructed bronchus. Thoracotomy and surgical removal was an option, but also carried a high mortality rate. However, in his first case series published in 1908, Chevalier Jackson reported 17 successful foreign body retrievals with zero mortality [12]. At this time, he also warned that “endoscopic ability cannot be bought with instruments” [13]. To aid in the dissemination of safe endoscopy and bronchoscopy he established a course in Pittsburgh at Western Pennsylvania Hospital. His achievement did not go unrecognized. At only 35 years of age, he was appointed chair of laryngology at Western Pennsylvania Medical College. Subsequently, he was appointed to the staff at 14 hospitals in Pittsburgh, immune to institutional non-compete policies, and patients from far and wide flocked to Pittsburgh to seek his aid. The magnitude of the impact of safe bronchoscopy cannot be understated. Through his efforts, Chevalier Jackson transformed the prognosis of foreign body aspiration from 98% mortality to 98% survival [2].

## Early days in Pittsburgh that shaped the man

Chevalier Jackson grew up in a bilingual (French and English) home with his two brothers, Morange Stanford Jackson (1873–1916), who would take over the family hotel, and Shirls Jackson (1870–1950), who would follow “Chev” into medicine as a Pittsburgh ophthalmologist. His father William Stanford Jackson (1829–1889), a stock-raiser, veterinarian and inn keeper, used to say “educate the eyes and the fingers,” a precept that young Jackson took to heart, and passed on to his surgical trainees later in life [14]. Jackson had no appetite for entertainment; achievement was his sole source of pleasure. From the age of four he began working with wood carving tools. As he got a bit older, he was more inclined to build skates, sleds, and canoes than to play with them. He went on to build intricate furniture, grandfather clocks, and a number of gavels used by several academic surgery societies.

**Fig. 5:**
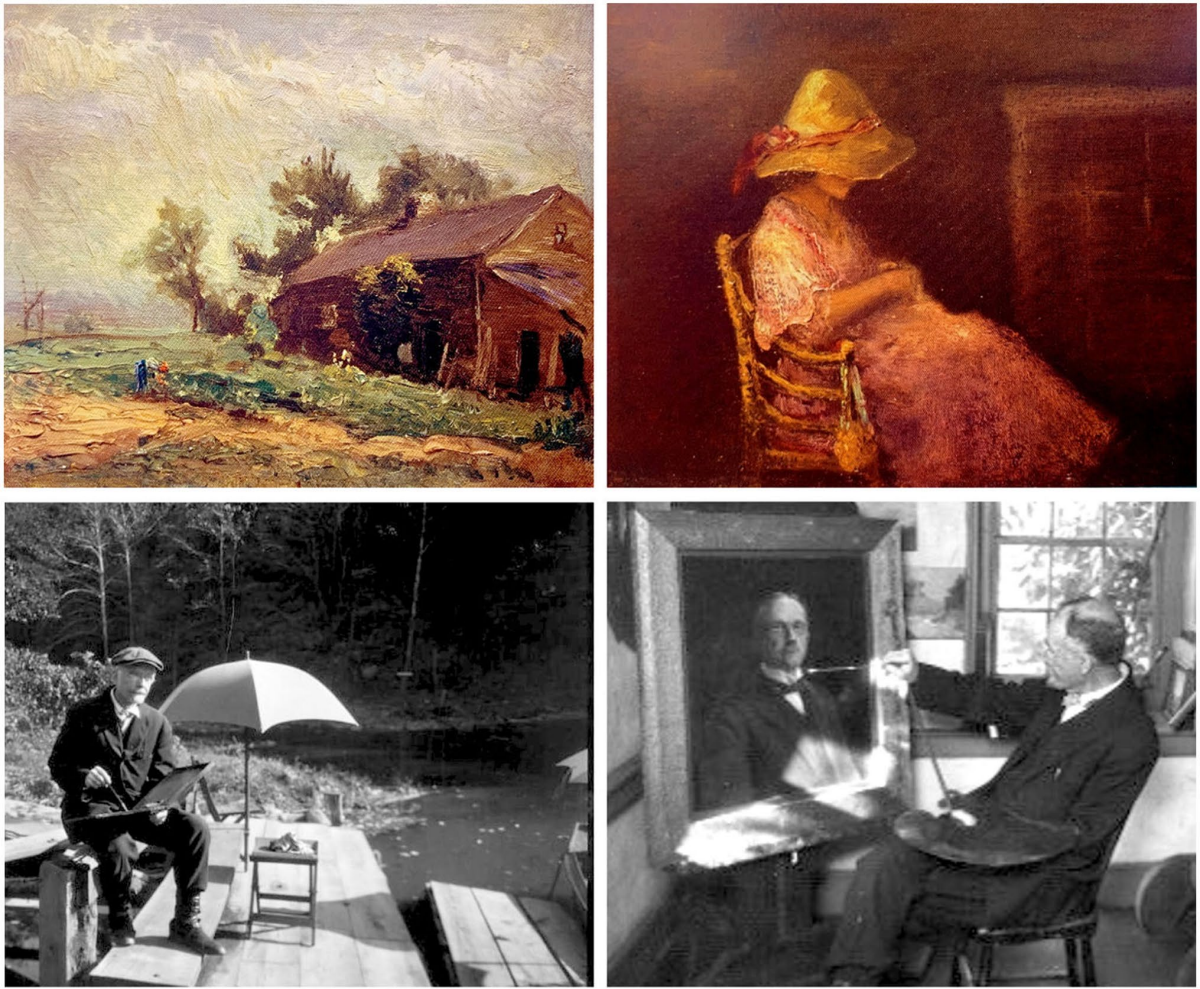
“The Old Man’s Garden” (1916) oil painting by Chevalier Jackson (Top Left). “Alice” (1912) by Chevalier Jackson (Top Right) an oil painting of his wife Alice Bennett White Jackson (1876–1957). Chevalier Jackson painting outside and inside his home at Sunrise Mill. *Photos obtained from The Life of Chevalier Jackson: An Autobiography. New York: The MacMillan Company; 1939*

**Fig. 6:**
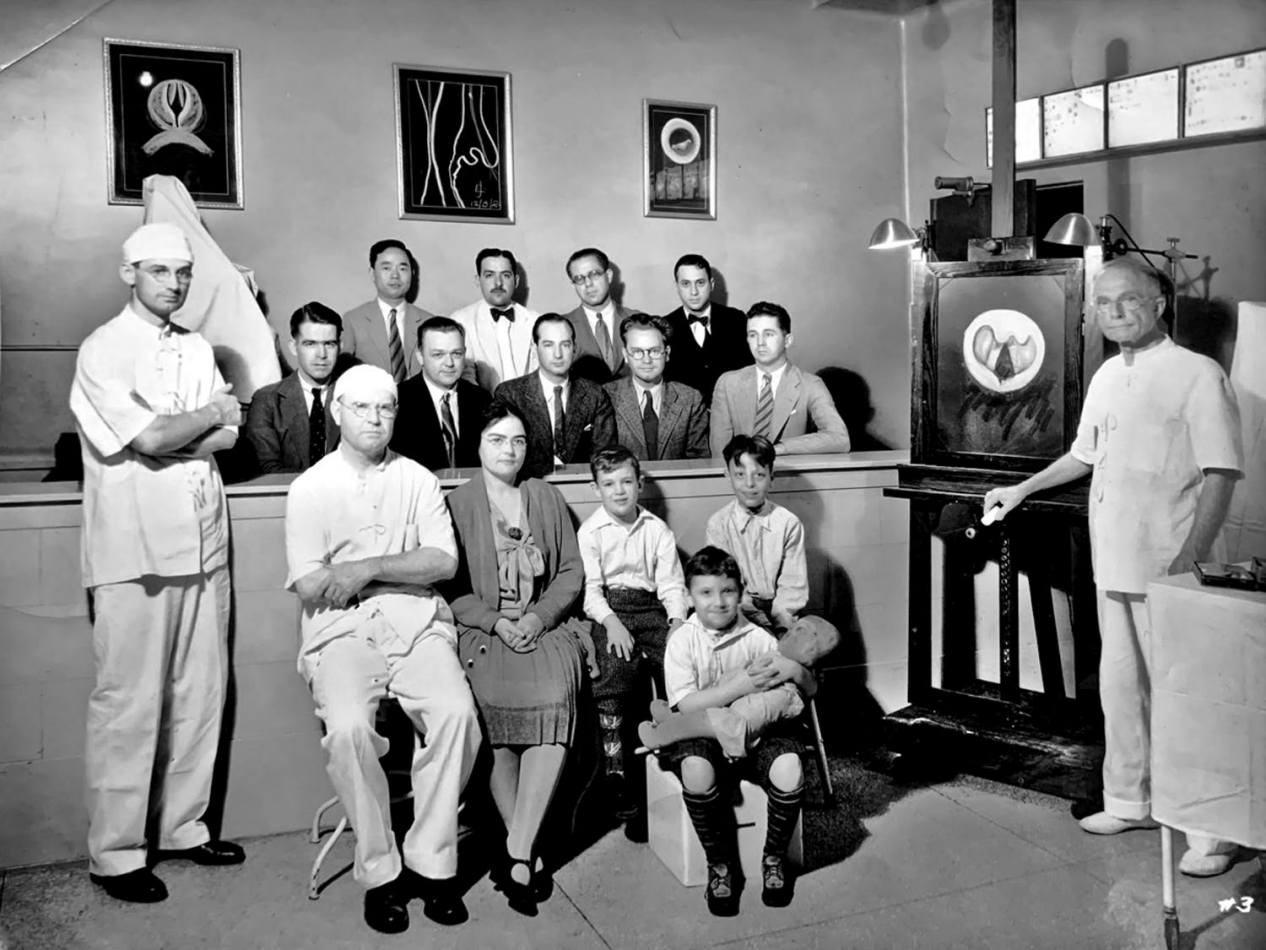
Chalk talk on tracheotomy. In the observation stand a group of physicians taking a bronchoscopic course. In the front from left to right: Chevalier L. Jackson, who followed his father into the field of esophagobronchoscopy; Dr. Penn Smith; Dr. Emily Van Loon, surgical trainee of Jackson who took over esophagobronchoscopy education at Woman’s Medical College after Chevalier Jackson; pediatric patients just treated holding a tracheotomy doll, which Jackson used to explain the procedure and teach the children to take care of their tracheotomies; and Chevalier Q. Jackson. *Photo obtained from Chevalier Jackson papers. 1890–1964. Located in: Modern Manuscripts Collection, History of Medicine Division, National Library of Medicine, Bethesda, MD; MS C 292*

Jackson grew up in Pittsburgh’s coal and oil territory during the rise of Andrew Carnegie’s steel empire. One day, the oil prospector who had struck an agreement with William Jackson to drill for oil on their land, reported that they would have to start a new well because the drilling tools had fallen 1500 feet down the well and were irretrievable. Chevalier, just a boy at the time, was intrigued by this impossible task, and designed and built an instrument to successfully retrieve the equipment, foreshadowing his illustrious career built on removing foreign bodies from tubes. Chevalier Jackson’s creative and artistic endeavors were not limited to carpentry and crafts; he was a skilled sketch artist and oil painter (Fig. 5**)**. However, he was most adept in the medium of chalk, which he used to expertly illustrate his endoscopic findings and to enliven his popular lecture series, “Chalk Talks” (Fig. 6). Despite “an obstinate case of right-handedness” Chevalier Jackson trained himself to be ambidextrous, which he used both as a ‘two-handed surgeon,’ and to illustrate his talks quickly bimanually, such as showing an asphyxiating patient morph into a happy smiling grateful patient with a few strokes of colored chalk. Truly, he was a nineteenth and twentieth century Renaissance man [2].

Empathy, composure and a desperate desire to ease the suffering of others were part of his character from childhood. Despite severe bullying, including the daily theft or destruction of his lunch, Chevalier Jackson never shed a tear for himself. He even begged for scraps on behalf of two children whose father favored spending his wages on alcohol over food for his family. However, Jackson was frequently brought to tears over his childhood helplessness to ease suffering or stop cruelty. Vicious dog fights, senseless beating of horses and general maltreatment of others at the hands of drunkards were a common feature of his early life and frequently left him sobbing uncontrollably. In one instance Jackson fled in tears from the scene of a father brutally beating his son for being too weak to pull the back brake of a horse drawn cart. Later that day Jackson came across the same cart and witnessed the boy fall from the wagon, landing head first in front of the rear wheel, killing him instantly. Jackson maintained his calm, commanded the horses to stop, attended to the mangled boy and went to fetch the doctor. Jackson shed no tears throughout, as the boy died immediately without suffering. It wasn’t until he heard the anguished cries of the boy’s mother that his helplessness to ease her suffering brought him to tears [2].

As an adult, Chevalier Jackson developed his ability to maintain a sincere and calm disposition, even in the face of brutal cruelty. When a woman approached him on the street to help stop her husband from beating her child to death, Chevalier Jackson employed his calm, soothing demeanor and skills at persuasion to de-escalate the situation with the husband so that he could attend to the child. Undoubtedly, it was this same patient and calm disposition that enabled Chevalier Jackson to successfully introduce per-oral endoscopic procedures at a time when reliable anesthesia was often unavailable. The child was asphyxiating on a coin she had hid from her father in her mouth so that the family could buy bread rather than liquor, incurring a strangling. An emergent tracheotomy was performed and the child recovered. Expert tracheotomy became a common procedure with the prevalence of diphtheria at the time. However, the advent of general anesthesia and its respiratory suppressive effects meant that many tracheotomies were performed in an uncontrolled and hasty fashion with high morbidity and mortality. In 1909, Chevalier Jackson became the first to fully describe a standardized approach, technique, and postoperative management regimen for tracheotomy. Jackson invented a number of instruments for airway management including the Jackson Laryngoscope, which was the first use of direct laryngeal visualization during intubation, and the Jackson Tracheostomy tube, still in use today [15].

## Advocate for patients, public health, and trainees

**Fig. 7:**
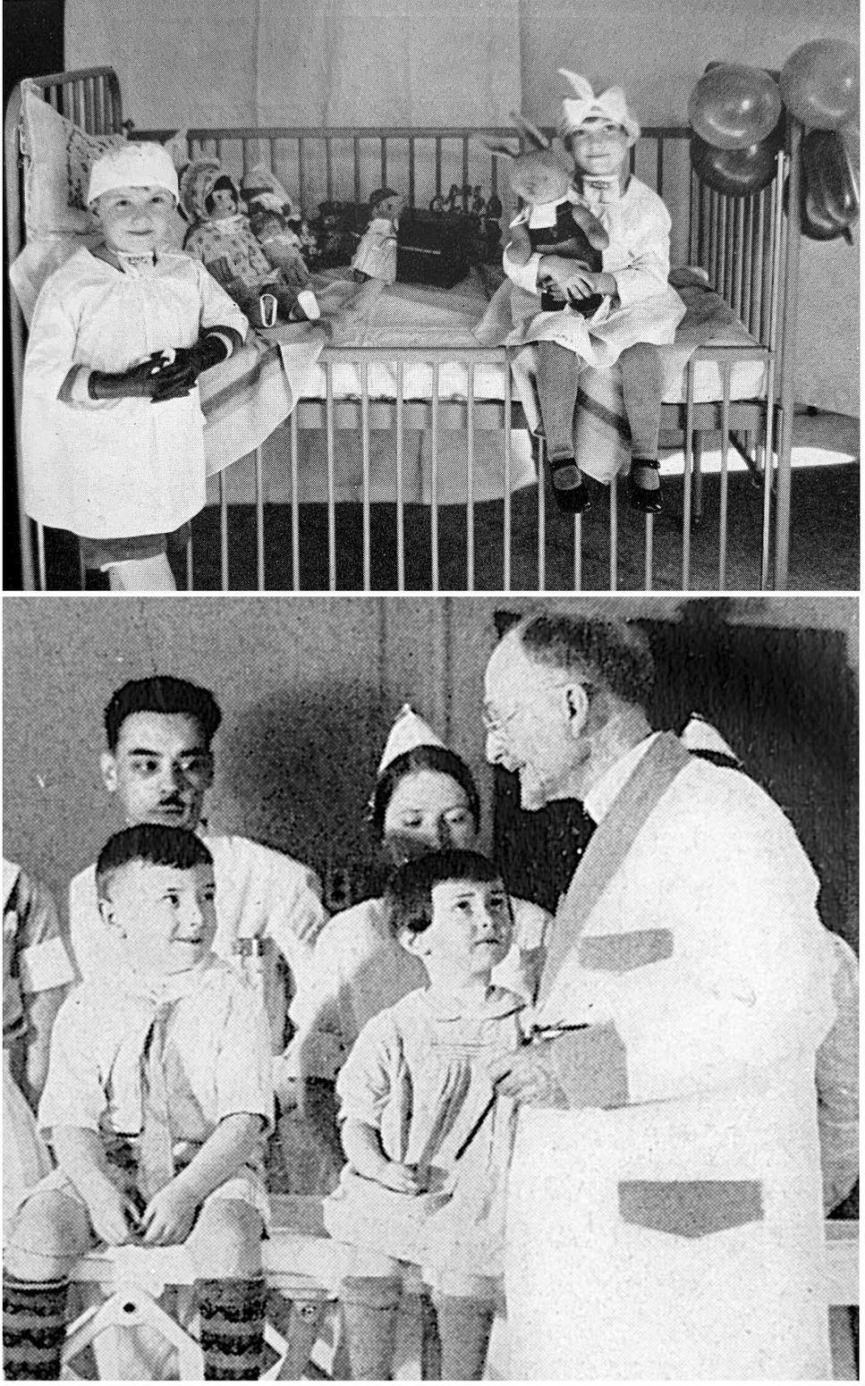
Chevalier Jackson was an early practitioner of what would become known as patient-centered care, encouraging patients to be involved in their care, even teaching them to self-dilate their strictures. Here he is explaining a procedure to the patients, scope in his left hand (Bottom). Patients at Temple University Hospital whose larynxes had been partially destroyed by diphtheria, requiring tracheotomy, are ‘playing doctor,’ imitating Chevalier Jackson, down to his classic stance in the operating room, and learning from toys and dolls, all of which have also had a tracheotomy (Top). *Photos obtained from Chevalier Jackson papers. 1890–1964. Located in: Modern Manuscripts Collection, History of Medicine Division, National Library of Medicine, Bethesda, MD; MS C 292. (Bottom) and The Life of Chevalier Jackson: An Autobiography. New York: The MacMillan Company; 1939. (Top)*

Patient advocacy was a foremost priority from the beginning of Chevalier Jackson’s career. He recognized the public health implications of recurrent tonsillitis in children. Headache, earache, and sore throat often made children irritable and prone to missing school. Jackson surmised that disease likely diminished academic performance. He investigated the matter himself, and found that many of the listless students, dismissed by their teachers as dull and apathetic, were actually suffering from hypertrophied and diseased tonsils. His tonsillectomy outcomes were measured in more than resolution of symptoms; the children demonstrated dramatic improvements in scholastic achievement after surgery. Chevalier Jackson swiftly brought this newly discovered public health issue to the attention of the head of Pittsburgh public schools and the chairman of the school board. Convincing the officials of the problem proved to be a challenge, requiring him to diligently document patient outcomes to develop a body of convincing evidence. After much toil, the elected officials were convinced of the problem, but were afraid of losing votes because of potential parental outrage at an officiated medical program in the schools. They failed to pass any meaningful policies but did agree to recommend parents of ailing children to see Dr. Chevalier Jackson, who treated them all, regardless of ability to pay. Before long, Chevalier Jackson’s clinic was overwhelmed with poor suffering children, and he was able to make a meaningful impact in each of their lives and his local community (Fig. 7) [2].

Chevalier Jackson’s public health advocacy did not stop at the local level. A frequent complaint in his office was a child with persistent dysphagia, odynophagia or inability to tolerate oral intake due to the consumption of lye. Jackson developed techniques for esophageal stricture dilation and endoscopic feeding tube placement, saving many children from a slow death at the hands of starvation and dehydration. However, he knew that prevention was better than treatment. At the time, lye was a very common household substance, and due to a lack of any warning labels and misleading ads for “harmless” dilutions, the substance was often ignorantly kept at floor level where it was easily accessible to children. Given its sugar-like appearance, caustic esophageal injury and its sequelae were not an uncommon occurrence. Chevalier Jackson could not idle faced with so many readily preventable injuries. Warning labels and a nationwide educational campaign were necessary. He began by petitioning the lye packers to voluntarily label their products. Individually the packers refused, claiming that warning labels would advantage unlabeled competition. Legislation was necessary, but was also strongly opposed by the industry, fearing labeling would hurt sales and advantage diluted preparations. Moral appeals to the benefit to humanity and the prevention of needless suffering of innocent children fell on deaf ears.

Jackson petitioned political leaders in the Pennsylvania House of Representatives, but again was met with strong resistance. He was warned that to pass any legislation he would need substantial financial support and spend years convincing a number of political groups and lobbyists to back the legislation, the most influential group being beer and liquor. In essence, he was strongly advised not to pursue the matter further. Chevalier Jackson was disheartened, but not defeated. At the time the vast majority of his caseload was charity, as he could not bear to cause a patient any financial hardship through treatment. As a result, he did not have the funds to run the necessary campaigns. He resigned to diligently document every caustic esophageal injury he encountered to build a body of evidence to make a case when he was able. After 15 years of practice and collecting evidence, Jackson had developed a steady stream of patients who could afford to pay without hardship, and the matter of warning labels could be tackled in earnest. He presented his evidence to the American Medical Association who promptly appointed him Chairman of a new Committee on Lye Legislation. He worked to fill the committee with representatives from all 48 states in the Union. The committee ran the sorely needed nationwide educational campaign through letters, photographs, interviews, lectures, and the like. Legislative success was slow, as bills were repeatedly defeated and put on a 2-year waiting period before they could be re-introduced. However, after 20 years of hard work, warning label laws were on the books in half the country and the movement was starting to get federal attention. It took a sum total of 25 years of campaigning, but on March 2nd, 1927, President Coolidge signed into law the federal Caustic Poison Act, which mandated poison and antidote labels on hazardous substances. Recognizing his contributions to this legislation, President Coolidge sent Chevalier Jackson the pen used to sign the bill along with the message “Thank you Doctor.” Chevalier Jackson’s commitment to public health has left an indelible mark on this nation, has prevented an untold amount of needless suffering, and lives on today as the Federal Hazardous Substances Act [16].

**Fig. 8:**
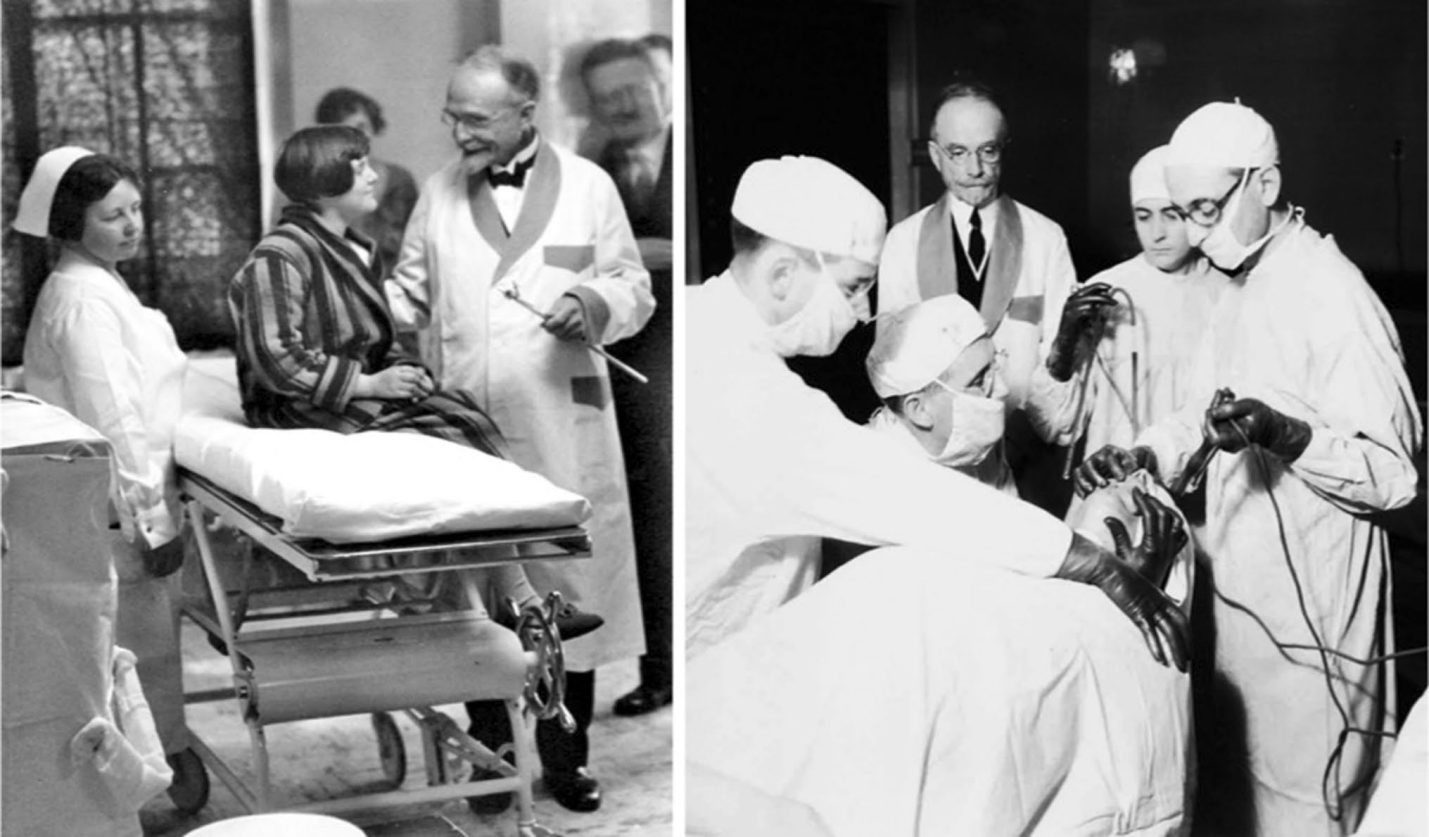
Chevalier Jackson meeting with a 10 year-old patient before endoscopy to explain the procedure and show the patient the endoscope, which is held in his left hand (Left). Jackson’s taught surgical trainees through graduated autonomy in the operating room, as shown here overseeing a trainee’s technique (Right).* Photos obtained from Chevalier Jackson papers. 1890–1964. Located in: Modern Manuscripts Collection, History of Medicine Division, National Library of Medicine, Bethesda, MD; MS C 292*

In addition to advocating for patients and public health, Jackson was an advocate for his multidisciplinary team, a concept that was highly unusual at the time. Many great surgeons, foreign dignitaries and visitors became indignant at being presented to the secretaries and nurses when they visited the prestigious endoscopy clinic at Western Pennsylvania Hospital. Chevalier Jackson had to explain to hundreds of offended visitors that everyone in his organization works with utmost professional deportment and was due respect. Furthermore, Chevalier Jackson believed that no one should develop an inferiority complex while working with him. To this effect, he strived to make each member of the team feel the importance of their contribution, whether it was the trainee who successfully removed a foreign body or the patient transporter who provided a nervous patient with a word of encouragement before a procedure. This commitment to fighting the inferiority complex was most true with his trainees. At the time, the prevailing culture was to train assistants merely to aid the surgeon. Chevalier Jackson felt an obligation to build the trainee’s confidence and an obligation to humanity to spread the gospel of safe endoscopy and bronchoscopy. As such, Chevalier Jackson believed in developing competence with the scope and the ego of the surgeon who holds it (Fig. 8). He aided his trainees with case reports and papers, fed them ideas, and sometimes gave them more credit than what was due. Several of his colleagues sent him letters over the years admonishing him for being too good to his assistants, who boasted of achievements they would never have been able to accomplish without Jackson. His response to these reprimands was that of joy, as he believed this boasting was evidence that his former trainees had become confident independent practitioners who would go on to spread the gospel of safe endoscopy and bronchoscopy [17].

## Champion of social justice and the role of women in medicine

Social justice and inclusion were at the foundation of Chevalier Jackson’s practice. He was known to say that a “patient is a patient” with race, religion, and gender only considered if relevant to the scientific problem. Throughout his career he used his national recognition and influence to create opportunities for women physicians and physicians of color, during a period of marked discrimination and adversity. When Dr. William Harry Barnes (1887–1945), the first African American to receive a medical school scholarship to the University of Pennsylvania, decided to specialize in laryngology, he found difficulty finding training in 1920s United States as a person of color. Yet Chevalier Jackson accepted him at his Endoscopy and Bronchoscopy Clinic, becoming his life-long mentor, and Barnes went on to become the first board certified African American in any specialty [18].

Chevalier Jackson had a lifelong respect for the status of women at a time when there was much abuse and maltreatment of women in homes and the workplace. Despite the daily bullying, witness to cruelty toward animals and exposure to brutality that permeated Chevalier Jackson’s early years, a woman was never a willing participant in any of it. This fact, in conjunction with the kindness of his schoolteacher, Miss Ward and the sisters of his catholic school, instilled in him a deep respect for women. His mother, Katherine Ann Morange Jackson (1836–1924) was also a strong influence who, although not formally trained as a physician, was so adept at administering medicines that he regarded her as “a better physician than [he] ever was” [2]. Throughout his professional life he was a passionate advocate for women physicians. His first assistant while he practiced in Pittsburgh was Dr. Ellen Patterson, who worked side by side with him at Western Pennsylvania Hospital through the development of endoscopy and bronchoscopy. During a bout of tuberculosis, which left Jackson bedridden for a month—time he used to write his groundbreaking book *Per Oral Endoscopy and Laryngeal Surgery*—it was Dr. Patterson whom he trusted to the run clinic. When Chevalier Jackson retired from clinical practice in Pittsburgh, Dr. Patterson became chief of the endoscopic clinic [19]. He then moved to Philadelphia, where he was appointed Special Lecturer at the Woman’s Medical College and gave regular and lively lectures to aspiring young woman physicians.

**Fig. 9:**
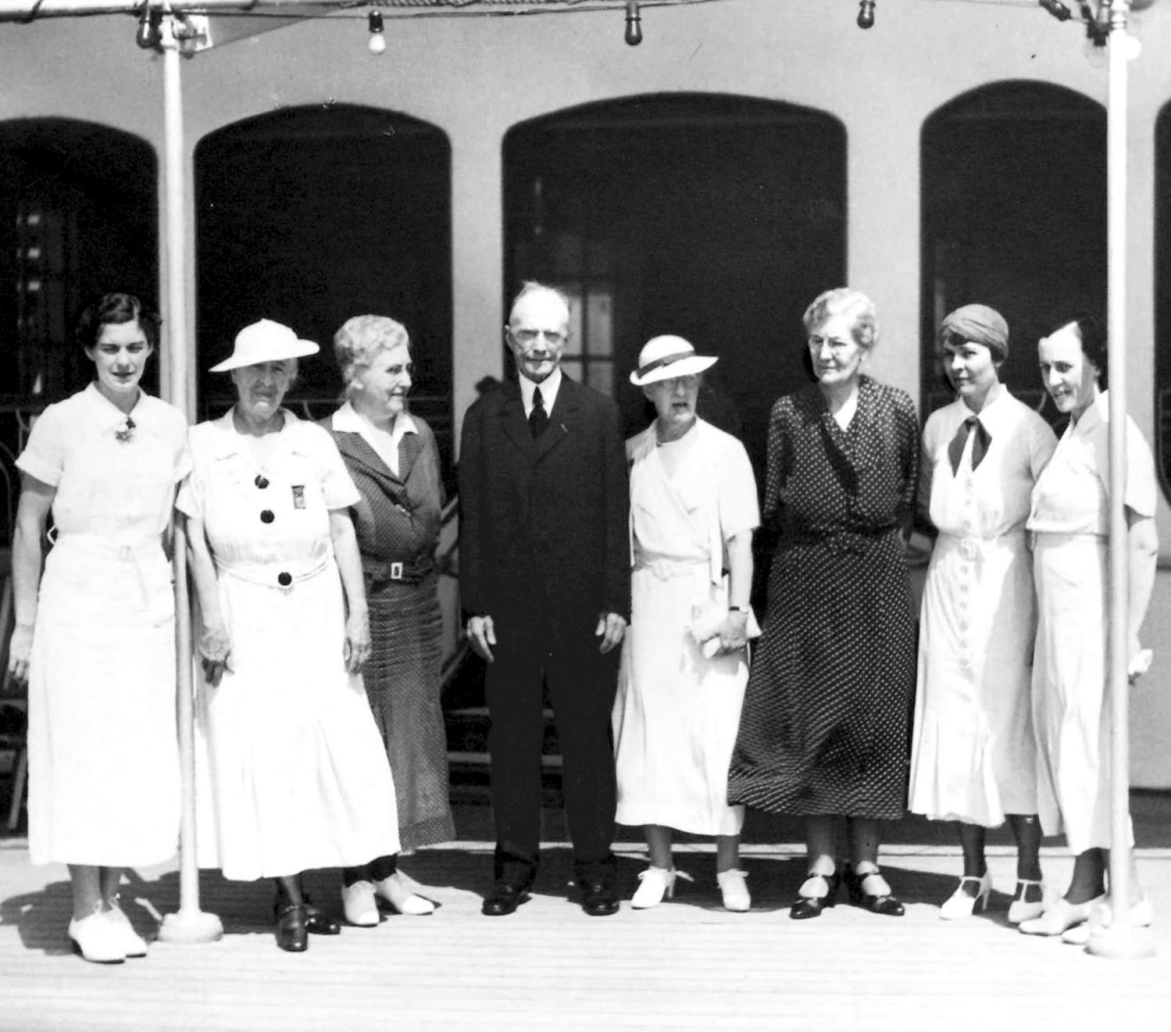
Sixth Pan American Medical Congress, Havana, Cuba, 1928. In order from left to right, Dr. Leone Cottrell; Dr. Grace Ritchie-England; Dr. Frances B. Tyson; Dr. Chevalier Jackson, President of the Woman’s Medical College of Pennsylvania; Dr. Ellen J. Patterson, Chief of the Pittsburgh Endoscopy Clinic; Dr. Lydia A DeVilbiss; Dr. Virginia Van Meter and Dr. Jeannette Cohen. *Photos obtained from The Life of Chevalier Jackson: An Autobiography. New York: The MacMillan Company; 1939*

Elizabeth Blackwell was the first woman in America to earn a medical degree, a mere 15 years before Chevalier Jackson’s birth, and the same year Woman’s Medical College of Philadelphia was founded. By the time Chevalier Jackson began his career, avenues for women in medicine were opening, but the road was not easy to follow. Many physicians complained that it was offensive, indecent, and disrespectful to have women in medical school. The general public felt it unbecoming and inappropriate for women to be involved in such intimate details of patients’ lives, and the sobriquet “female physician” was more often used to refer to a back-street abortionist than a woman with a Doctor of Medicine [20]. Chevalier Jackson abhorred and actively opposed these misogynistic sentiments. In 1934, he decided that he needed to operate more often at the Woman’s Medical College and petitioned the board of trustees to establish an endoscopy and bronchoscopy clinic. They created a chair of Bronchoscopy and Esophagoscopy position with Chevalier Jackson as professor, and regular instructional clinics began. Chevalier Jackson was ever cordial and respectful of his assistants from the Woman’s Medical College, sure to always address them by the title they had earned: Doctor. On more than one occasion he was invited to give the commencement speech at Woman’s Medical College, during which he always closed with “if, in any of the four corners of the earth to which you will scatter, you need someone to vouch for you, or lend a helping hand, write to me with Woman’s Medical College in your signature.”[21] He held true to his word, as a few years later a young physician contacted him about an unsuccessful appointment to a New York hospital residency that had never before taken a woman. He telephoned the chief of the service at the hospital and the young physician promptly became the first woman in the program.[22] In his long career decorated with countless awards and leadership positions, Chevalier Jackson only ever served as president of one educational institution, Woman’s Medical College (1935–1941) (Fig. 9). In one of his speeches as president he explained that it had been his “solemn obligation all [his] professional life to help in every way possible every woman physician.”[21].

## A man of utmost moral character and commitment to humanity

**Fig. 10:**
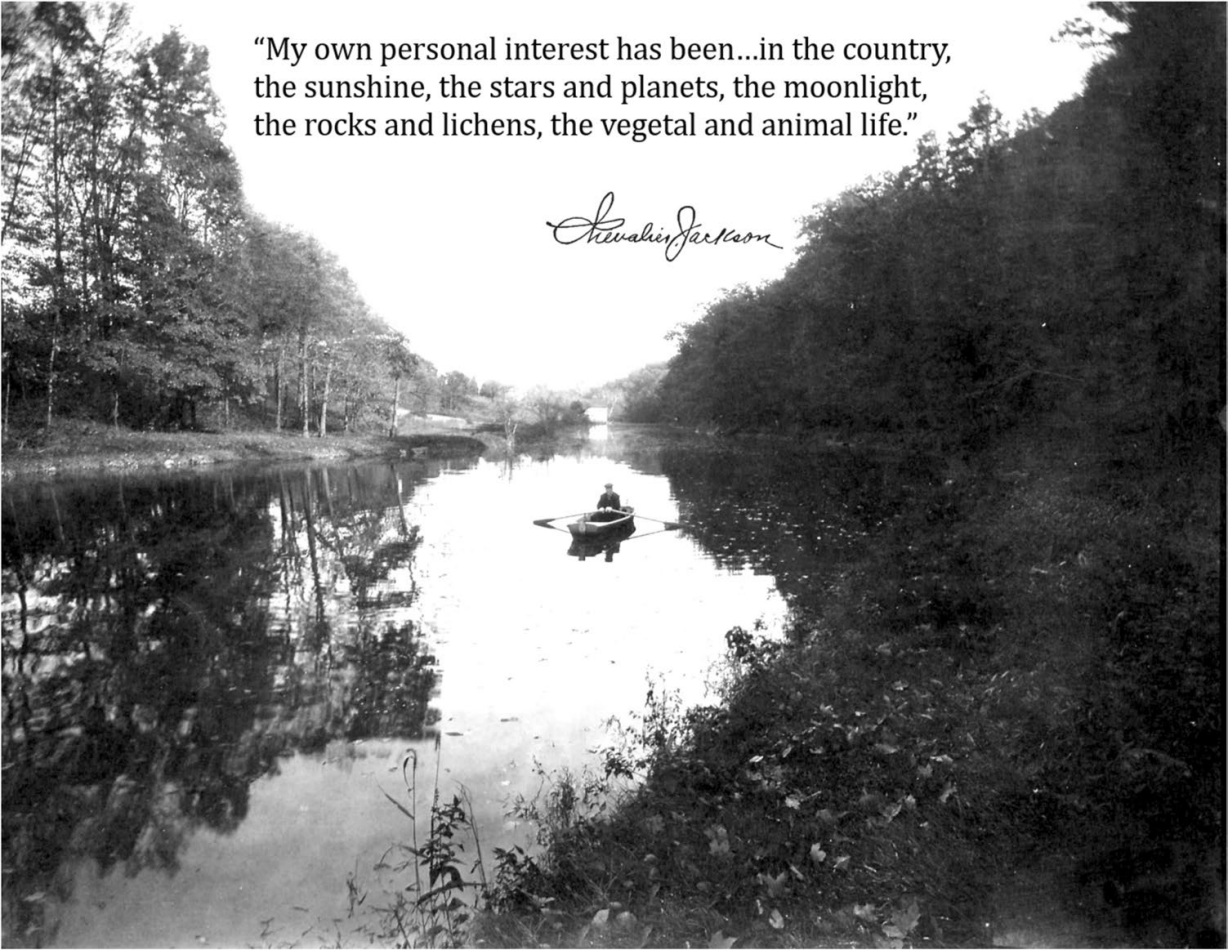
Chevalier Jackson on a homemade boat in the pond at Sunrise Mill. The quote from his autobiography with his handwritten signature describes his deep love of nature and desire to live a peaceful life at home. *Photos obtained from The Life of Chevalier Jackson: An Autobiography. New York: The MacMillan Company; 1939*

Chevalier Jackson’s list of accomplishments are far too numerous to detail, but perhaps the greatest testament to the man’s character was that he was never motivated by money, prestige or political power. Rather he was motivated by pure and sincere curiosity and a moral obligation to serve humanity. Socially, Chevalier Jackson was a consummate introverted recluse, most content to stay at home tinkering, toiling, and watching the squirrels. His station earned him invitations to countless social engagements, all of which he refused. Yet the tremendous impact of the development of endoscopy and bronchoscopy compelled him to vocally discuss the science at every opportunity. Despite profound homesickness, he traveled thousands of miles on horseback, by automobile and by sea to disseminate his techniques and technology, and to advocate for patients, public health policies and young surgeons. He paid for all his travel from his own pocket, despite the fact that for most of his career his clinic was 95% charity. He also refused to patent his inventions so as to more freely promote their adoption to service patients in need and was never paid for any of his numerous leadership positions. After 30 years of practice in Pittsburgh, he was given the opportunity to open a new clinic in Philadelphia as a professor at Jefferson Medical College. This was an opportunity for greater dissemination in the city with the most medical colleges in the country at the time, but meant leaving his lifelong home and his elderly mother. However, she encouraged him to take the position, because the impact of endoscopy and bronchoscopy would far outlive either of them. He purchased Sunrise Mill (a historic gristmill and sawmill, now museum) just outside Philadelphia and commuted into the city (Fig. 10). Once the Jefferson clinic was self-sufficient, he started another at University of Pennsylvania, then Graduate Medical College, then Temple University Hospital and finally Woman’s Medical College. Each time he started a new clinic he resigned from his previous prestigious position. However, his resignations were all refused, which is how Chevalier Jackson came to sit as department chair at all 5 Philadelphia medical colleges simultaneously, a unique feat, which is unlikely to ever be repeated [2].

## Conclusion

Chevalier Jackson was an extraordinary surgeon, pioneer in the profession, expert educator, public health activist and advocate for diversity and inclusion in medicine. Jackson’s legacy is a testament to what a surgeon can accomplish when driven by compassion, integrity and innovation with persistence and refusal to be deterred. He launched his career at Western Pennsylvania Hospital, where he developed the endoscope, and made many other extraordinary contributions to the surgical profession. Therefore, in honor of this surgeon of immense character, whose pioneering spirit and commitment to service continue to inspire us, the Esophageal Institute at Western Pennsylvania Hospital has founded the Chevalier Jackson Esophageal Clinical Research Fellowship. This fellowship will help foster a new generation of healthcare professionals inspired by Jackson’s legacy, who are committed to upholding the highest standards of excellence, inclusivity and advancement of the surgical profession. We are proud to be part of this legacy, and hope that the Chevalier Jackson Research Fellowship will be a fitting tribute to his memory and his pioneering spirit.
